# Lifetime Costs and Outcomes of Repair of Tetralogy of Fallot Compared to Natural Progression of the Disease: Great Ormond Street Hospital Cohort

**DOI:** 10.1371/journal.pone.0059734

**Published:** 2013-03-22

**Authors:** Rachael Maree Hunter, Mark Isaac, Alessandra Frigiola, David Blundell, Kate Brown, Kate Bull

**Affiliations:** 1 University College London, London, United Kingdom; 2 Great Ormond St Hospital NHS Foundation Trust, London, United Kingdom; 3 Leeds School of Medicine, Leeds, United Kingdom; Sapienza University of Rome, Italy

## Abstract

**Background:**

Tetralogy of Fallot is a congenital heart disease that requires surgical repair without which survival through childhood is extremely rare. The aim of this paper is to use data from the mandatory follow-up of patients with Tetralogy of Fallot to model the health-related costs and outcomes over the first 55-years of life.

**Method:**

A decision analytical model was developed to establish costs and outcomes for patients up to 55 years after diagnosis and first repair of Tetralogy of Fallot compared to natural progression. Data from Adult Congenital Heart Disease (ACHD) centres that follow up Tetralogy of Fallot patients and Great Ormond Street Hospital (GOSH), London, United Kingdom (UK) medical records was used to establish the cost and effectiveness of current interventions. Data from a Czech cohort was used for the natural, no intervention condition.

**Results:**

The average cost per patient of a repair for Tetralogy of Fallot was £26,938 (SE = £4,140). The full life time cost per patient, with no discount rate, was £65,310 (95% CI £64,981–£65,729); £56,559 discounted (95% CI £56,159–£56,960). Patients with a repair had an average of 35 Quality Adjusted Life Years (QALYs) per patient over 55 years undiscounted and 20.16 QALYs discounted. If the disorder was left to take its natural course, patients on average had a total of 3 QALYs per patient with no discount rate and 2.30 QALYs discounted.

**Conclusion:**

A model has been developed that provides an estimate of the value for money of an expensive repair of a congenital heart disease. The model could be used to test the cost-effectiveness of making amendments to the care pathway.

## Introduction

Tetralogy of Fallot is the commonest cyanotic heart condition and was one of the first congenital heart diseases of any complexity to be repaired [1]. The condition affects about 0.31/1000 live births [2], with approximately 250 repairs of Tetralogy of Fallot being undertaken annually in the United Kingdom (UK) [3]. Survivors of the surgery in its ground-breaking era form a cohort of patients with a ‘new disease’ whose natural history they are themselves delineating.

Currently, the diagnosis of Tetralogy of Fallot may be achieved through pre-natal ultrasound screening or emerge at the time of an emergency presentation in infancy or during investigation of a murmur or an intercurrent illness. All patients require surgical repair without which survival through childhood is extremely rare. The repair is not fully ‘corrective’, primarily because obstruction in the area between right ventricle and pulmonary artery must be relieved, often sacrificing pulmonary valve function. Service standards require lifetime follow up for all Fallot patients [4]. For some, surgical revision of the right ventricular outflow tract area is required later – most usually a pulmonary valve replacement (PVR). Though for an individual patient, the future need for PVR is hard to judge in early childhood, cohort studies are emerging that permit prediction of the proportions requiring later revision [5].

Achieving early surgical survival required a demanding learning curve – 30-day mortality rates were commonly around 25% in the 1960s and are currently around 10-times lower. Because early outcomes have been good for many years, the great majority of children currently treated leave ‘childhood’ services fit to face adult life [6]; they are looked after by a network of specialist Adult Congenital Heart Disease (ACHD) centres. With the benefit of a primary data source - a cohort of Fallot patients 1964–2009 - our aim was to estimate the first 55 years health-related costs and outcomes for patients with Tetralogy of Fallot born now and managed by current standards.

## Methods

Decision analytic models use the best available information from a range of sources to estimate the costs and consequences of a particular intervention or policy, providing they take into account the uncertainty associated with the variables contained in the model [7]. We developed a decision analytic model to calculate the health outcomes and costs of surgical repair of Tetralogy of Fallot over a 55 year time horizon. The primary data source is a complete consecutive list of all UK patients having repair of uncomplicated Tetralogy of Fallot at Great Ormond Street Hospital (GOSH), London, UK, from February 1964 to January 2009. Secondary data sources have been used where necessary.

### Great Ormond Street Hospital Cohort

Data was available on all 1085 UK patients who had a repair of Tetralogy of Fallot at GOSH from February 1964 to January 2009. The GOSH medical records provided date and age at repair, dates of any PVRs and dates of death if these events occurred in childhood. Demographic data was also linked to NHS numbers, providing information about deaths since 1996 and collaborators from each ACHD centre used local databases to provide dates of any PVRs done for patients over the age of 16. The 46 patients lost to follow-up were censored on the last date of follow-up recorded.

Parents of current patients are asked to give written consent for patient details to be collected and stored for audit and research purposes. The NHS Research Ethics Committee approved the collection and storage of resource use information from medical records from unconsented patients including the use of NHS numbers for later tracking.

### Survival and Reoperation-free Survival

Natural survival for Tetralogy of Fallot patients with no repair was estimated from Samenek (1992) where data on incidence of congenital heart disease (CHD) was combined with date of death to determine the distribution of age at death for all children with CHD in Central Bohemia, Czechoslovakia, over a 27 year period [8]. No intervention for Tetralogy of Fallot was available at the time hence the observational study provides data on the attrition of the disease without intervention. This information was used to construct a parametric (Weibull) survival model.

The 1085 patients in the GOSH cohort data provided the inputs for the parametric models (Weibull) we used to summarise survival from birth, with and without PVR. This was done in line with methodology set out in Briggs et al [9].

All analyses were conducted in Stata v10.

### Resource Usage: Frequencies

Age specific frequencies of routine health interventions were based on data from the cohort as follows:

### Resource Usage: between Birth and Open Heart Repair

We used data from patients born since 2000 to populate the model for this phase. In clinical terms, this period includes the phase of presentation and diagnosis, preoperative investigations and surveillance. Of the 214 patients in the cohort born in this era, 30 were selected at random and the in-patient and outpatient events and investigations each received were aggregated from their clinical records. Presentation was coded as ‘antenatal diagnosis’, ‘presentation with emergency hospital admission’ or ‘diagnosis achieved in an out-patient context’. Hospital lengths of stay, diagnostic and assessment investigations and numbers of out-patient visits were aggregated for these patients to represent the current pre-operative costs. There were no deaths in this interval.

### Resource Usage: Admission for Open Heart Repair

Actual data from the same 30/214 patients were used to estimate multipliers for the current costs for the open heart operation. The data used included the cost of the operation, length of stay, ward type and any major postoperative complications.

### Resource Usage: between Open Heart Repair and 10 Years of Age

Data from the same patients repaired since 2000 and who have not already required a PVR were used to estimate the current costs of postoperative surveillance, largely outpatient visits and investigations up to age 10. Interventions, whether catheter-based or surgical but which did not constitute a PVR were aggregated and the counts used as multipliers for these events.

### Resource Usage: Age 10–20 and Subsequent Decades

Clinical events (primarily out-patient visits and investigations) from age 10–20 of all twenty-one surviving operated patients born in 1990 were aggregated and rendered as an annual estimate of the clinical event rate for patients aged 10–20. We assumed that the rate of clinic visits and investigations would remain at rates similar to those at ages 10–20 for subsequent decades. Re-interventions short of PVR were aggregated as before.

### Cost of PVR

Data from the same 30/214 patients repaired since 2000 were used to estimate multipliers for the current costs for PVR. Data used related to length of stay and major postoperative interventions.

### Resource Usage: Costs

Based on the above cohorts we calculated the average resource use from birth until first repair, per decade and per PVR for patients who have a second repair. Per decade costs were divided by 10 to obtain a weighted cost per patient per year. All costs were in British Pounds (£) and 2010/2011 values. Unit costs for interventions and investigations other than first repair, PVR and Extracorporeal Membrane Oxygenation (ECMO) were derived from reference costs 2010/2011 [10].

The cost of the open repair was obtained from the GOSH patient level costing system for 30/214 Tetralogy of Fallot patients. This was divided by the 2010 GOSH market forces factor to obtain a UK average cost of repair. It was assumed that a PVR operation itself would have the same cost as the primary repair operation, although additional costs for length of stay and additional procedures were handled separately.

The cost of ECMO was obtained from Brown et al, and was estimated at £10,539 per day over 6 days for a total cost per ECMO of £75,126, accounting for inflation [11].

### Quality of Life

From the complete consecutive list, 50 survivors (10 from each surgical decade 1960’s to 2000’s) were chosen at random to receive a Quality of Life questionnaire; these patients (age 4–55) were assumed to be representative of survivors generally. In the absence of a generic quality of life instrument applicable across child and adult populations, the PEDSQL was administered to those age 1–18 and WHOQOL Bref to the adults.

The outcome measure used in the model was quality adjusted life years (QALYs). QALYs represent both the quality and quantity of health related quality of life, quality being measured by utility scores. A utility score of 1 represents perfect health and a utility of 0 death; negative values, representing states worse than death, are possible. QALYs are the recommended outcome for use in economic evaluations in the UK as they are a common unit that allow for comparable decisions about resource allocation across different diseases. In England, to ensure consistency of approach, the National Institute for Health and Clinical Excellence (NICE) recommends that utility scores used to calculate QALYs are calculated from the EuroQol 5D (EQ-5D), a 5 domain 3 level tool, and an algorithm developed by Dolan which is based on valuations of a selection of the 243 potential EQ-5D health states by 3,995 members of the general population [12].

There are no utility scores to calculate QALYs available for the WHOQOL. Instead we used a methodology similar to that used by Al Ruzzeh et al [13] to calculate utility scores from the WHOQOL; responses to WHOQOL questions which are similar to the EQ-5D dimensions were used to calculate utility scores. The utility values for the 5 level cross walk EQ-5D value set [14] were used so there was no need to covert the 5 levels of the WHOQOL to 3.

Similarly there are no utility scores available for PEDSQL, so the same methodology was used again but instead the questions are similar to those of the Child Health Utility 9D (CHU9D), for which utility scores are available [15]. We used the CHU9 algorithm applied to PEDSQL to calculate average utility for patients under 18.

As there is no quality of life (QoL) data available for the natural progression, for the ‘no surgical repair’ comparator cohort a random number between 0 and 1 was chosen to represent their utility scores.

### Description of Model

**Figure 1:**
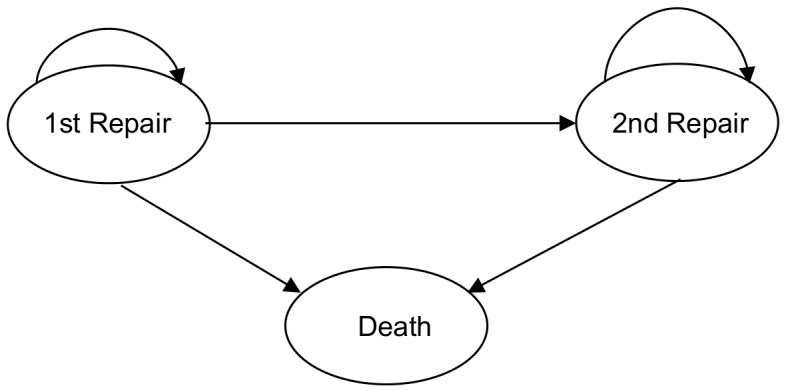
Markov model of health states for repair cohort.

The model used a time dependent Markov model, as described above, to simulate time to death from first repair, time to PVR and time to death from PVR over 55 years (see figure 1). The hypothetical population in the model is assumed to have the same characteristics as the cohort of patients described above.

The model was built in Microsoft Excel 2010 and is available as supporting material (Model S1).

### Cost Effectiveness Analysis and Probabilistic Sensitivity Analysis

Cost effectiveness was calculated as the incremental cost, in British Pounds (£’s), per QALY gained from surgical repair of Tetralogy of Fallot compared to natural progression, no repair. This provides a summary of the incremental, or extra, costs of the surgical repair plus all heart related follow up appointments, divided by the incremental, or extra, benefits.

A probabilistic sensitivity analysis (PSA) was conducted to calculate the cost effectiveness of surgical repair using the net monetary benefit approach as set out in Briggs et al [9]. These values were used to generate a cost effectiveness acceptability curve to determine the probability that surgical repair of Tetralogy of Fallot is cost effective for a hypothetical willingness to pay for each additional QALY gained for values of between £0 and £20,000. Results are based on 10,000 simulations. We provided results for undiscounted and discounted models. The discount rate was randomly varied between 0% and 6% in line with NICE guidance [12].

## Results

### Costs

A summary of the average resource use per patient is reported in Table 1. The average cost per patient of the admission including repair of Tetralogy of Fallot was £26,938 (SE = £4,140). In 2010 GOSH had a market forces factor of 1.18 [16]. After applying the market forces factor, the average UK cost for a repair was calculated as £22,829. The mean full life time cost per patient, with no discount rate, is £65,310 (95% CI £64,981–£65,729) and £56,559 discounted (95% CI £56,159–£56,960).

**Table 1:** Average resource use per patient: Mean (SE).

Resource	Birth to first repair	First decade following repair	Resource use per decade (2+)
	N = 30	N = 21	N = 14
Outpatient appointment	3.27 (0.42)		
ECHO	6.07 (0.4)	0.62 (0.02)	2 (0.62)
MRI	0.13 (0.1)	0.33 (0.14)	1.93 (0.41)
ECG	1.43 (0.2)	0.24 (0.15)	3.21 (0.75)
24 hr ECG	0.13 (0.06)	0.1 (0.07)	1.57 (0.37)
Ultrasound	0.73 (0.3)		
X-ray	8.77 (1.3)	0.14 (0.14)	2.5 (0.99)
ECMO	0.03 (0.03)		
Preliminary procedure	0.17 (0.03)		
Other Cardiac Surgery		0.05 (0.05)	0.07 (0.07)
Exercise lab		0.14 (0.08)	1.29 (0.41)

### Mortality and Quality of Life

**Figure 2:**
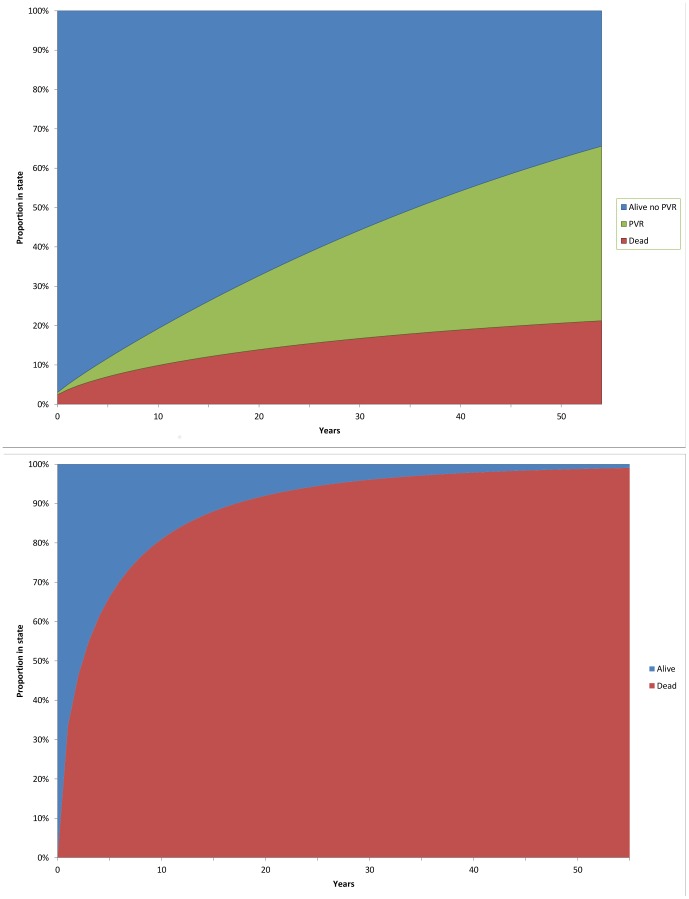
The changing proportion over time between 0 and 55 years in which patients are in 3 exhaustive and mutually exclusive states: ‘dead’, ‘alive without PVR’ and ‘PVR’. A) With repair; B) Natural progression, no repair.

Figure 2 provides a summary of the proportion of patients in each health state over time for the two models. The utility score for patients under 18 with a repair was 0.83 (SE = 0.031) and 0.72 (SE = 0.037) for adult patients. Patients with a repair had an average total of 35 QALYs with an average total of 3 QALYs for patients with no repair.

### Incremental Cost per QALY Gained

**Figure 3:**
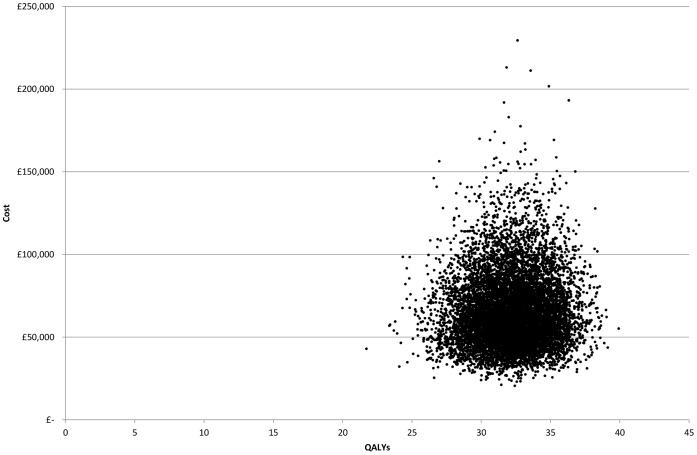
Cost-effectiveness plane – cost per Fallot patient over 55 years graphed against the QALY gained compared to natural progression –10,000 simulations.

**Figure 4:**
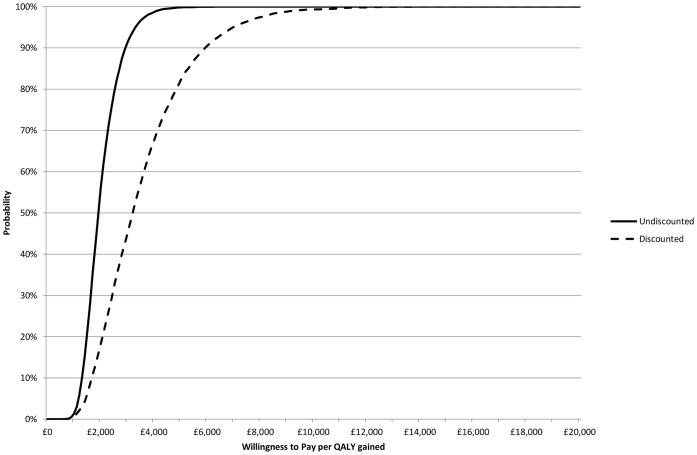
Cost effectiveness acceptability curve comparing repair of Tetralogy of Fallot with no repair: undiscounted (solid line) and discounted (dashed line).

The mean cost per QALY gained over 10,000 simulations was £2027 without discounting (£3168 discounted). All simulations fall into the north-east quadrant of the cost-effectiveness plane, in that all simulations result in more QALYs but also cost more (figure 3). Based on a willingness to pay of £20,000 per QALY gained there is a 100% chance that open repair of Tetralogy of Fallot is cost effective compared to doing nothing (figure 4).

**Figure 5:**
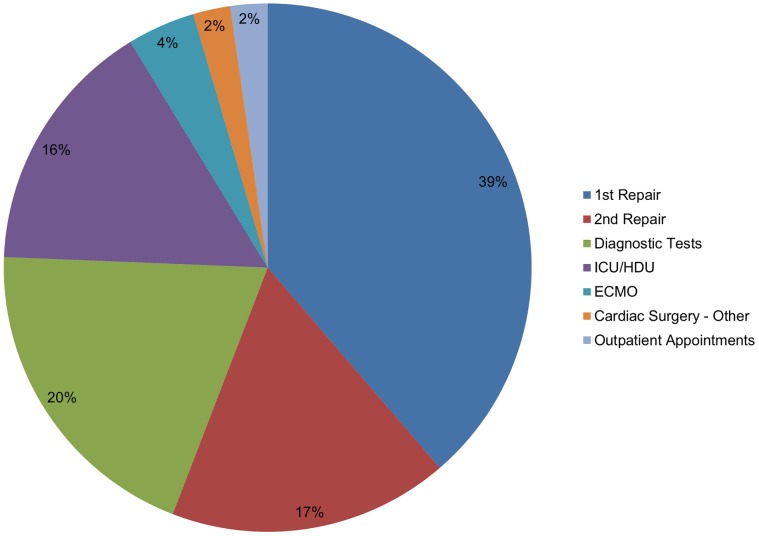
Breakdown of health care costs for Tetralogy of Fallot patients from birth to age 55: undiscounted.

Figure 5 shows the proportions of overall costs attributable to the surgery, inpatient stays in intensive care and high dependency units, outpatient appointments and investigations.

## Discussion

This model provides information on the first 55 years costs of the common congenital heart problem Tetralogy of Fallot and compares it to the additional gain in QALYs. The first 55 years cost of a Fallot patient to the NHS is approximately £65,310. This cost is outweighed by the additional years of life gained in reasonably full health. Ungerleider et al [17] published estimates of hospital costs of repairing Tetralogy of Fallot, including an evaluation of the alternatives of primary repair versus repair after preliminary palliative procedures. However we believe this is the first time a full cost effectiveness evaluation has been attempted in the field of childhood heart disease.

### Limitations

Surgery for Tetralogy of Fallot only emerged in the 1960’s, hence the postoperative “natural history” beyond age 55 is unknown; Fallot patients’ underlying biology makes it conceivable that heart related costs could escalate in later life. The natural progression, or no intervention, approach had only limited data from historical observational studies where no intervention was available, as it would be unethical to collect further information currently. None the less, this model presents confirmation of the benefit of expensive childhood interventions that significantly improve survival and quality of life of patients. Quality of life of surgical patients would need to decrease by 85% before the added life expectancy provided no additional benefit because of poor quality of life, even assuming perfect health for the ‘do nothing’ approach.

The results for both the discounted and undiscounted models have been presented; it is arguable which should be used in a context where surgery is offered to an infant as an ‘investment’ for their adult life. For patients living to the age of 55, using a discount rate of 3.5%, a year of life lived in perfect health is worth less than half a QALY. This seems to undermine the purpose of the intervention, which is to increase the chance that the patient reaches adult life in close to full health. This is supported by the evidence that people would like to give greater weight to younger people to represent the additional potential life they have to lose or gain. Trying to formalize the measurement of this though has proved difficult [18].

### Conclusions

Repair of Tetralogy of Fallot is a worthwhile investment for the health care system given the QALYs gained. This model could be used as a baseline to test the cost-effectiveness of changes to the care pathway for Fallot babies. Such changes could include improving screening procedures, changes in conduct of the primary repair with a view to changing the long term incidence of PVR and trials of the impact of elective PVR in adult life – all substantive questions for the congenital heart disease community.

## Supporting Information

Model S1 (XLSM)
